# Human Milk Lipids and Small Metabolites: Maternal and Microbial Origins

**DOI:** 10.3390/metabo13030422

**Published:** 2023-03-13

**Authors:** Lisa F. Stinson, Alexandra D. George

**Affiliations:** 1 School of Molecular Sciences, The University of Western Australia, Perth 6009, Australia; 2 Metabolomics Laboratory, Baker Heart and Diabetes Institute, Melbourne 3004, Australia

**Keywords:** human milk, metabolome, lipidome, microbiome, bacteria, synthesis, transport

## Abstract

Although there has been limited application in the field to date, human milk omics research continues to gain traction. Human milk lipidomics and metabolomics research is particularly important, given the significance of milk lipids and metabolites for infant health. For researchers conducting compositional milk analyses, it is important to consider the origins of these compounds. The current review aims to provide a summary of the existing evidence on the sources of human milk lipids and small metabolites. Here, we describe five major sources of milk lipids and metabolites: de novo synthesis from mammary cells, production by the milk microbiota, dietary consumption, release from non-mammary tissue, and production by the gut microbiota. We synthesize the literature to provide evidence and understanding of these pathways in the context of mammary gland biology. We recommend future research focus areas to elucidate milk lipid and small metabolite synthesis and transport pathways. Better understanding of the origins of human milk lipids and metabolites is important to improve translation of milk omics research, particularly regarding the modulation of these important milk components to improve infant health outcomes.

## 1. Introduction

As the first food for infants, human milk is a unique biofluid that provides the infant with all early-life requirements to thrive, including nutrient and bioactive factors. A wealth of evidence demonstrates that breastfeeding protects infants from acute and chronic illnesses and promotes the healthy development of various body systems [1,2,3,4,5]. Importantly, human milk forms the third arm of the mother-infant-milk triad [6], with human milk composition underpinning the complex relationship between mothers and infants. Various components of human milk contribute to healthy development and growth of the infant, including immune proteins, hormones, cytokines, leukocytes, microRNAs, macro- and micronutrients, microbiota, and stem cells [7,8,9,10]. Among the multitude of human milk components, lipids and small metabolites are of particular interest due to their likely importance in infant development, and the relative lack of research regarding their presence and function in milk [11,12].

Lipids are a major component of human milk, secreted as milk fat globules. These tri-layer structures deliver triacylglycerols, phospholipids, gangliosides, and many other lipid classes, to the infant [13]. The lipid portion of human milk is relatively complex, with wide intra- and inter-individual variation [14]. Although frequently thought to originate directly from maternal diet, the link between human milk lipids and diet is complex, with diet alone unable to account for the entire milk lipidome. Indeed, mammary tissue has significant lipogenic capacity. Human milk lipid composition is therefore a combination of incorporation from circulation and de novo synthesis. A number of human milk lipids have been linked to health and growth in early life. This includes many individual fatty acids, such as palmitic acid, which is present in higher abundance in human milk consumed by healthy infants compared to those who have cold-like illness [15,16,17]. The antimicrobial functions of human milk fatty acids are well known [17]. Additionally, a lower omega six to omega three (*n*−6:*n*−3) fatty acid ratio has been associated with increased lean body mass early in life and thus lowered risk of obesity [18]. Given the known importance of lipids in adult health and disease, human milk lipids have significant potential to modulate infant health and to program life-long health. A better understanding of the human milk lipidome will thereby aid in prediction or prevention of chronic health conditions [11].

Human milk also contains a multitude of small metabolites [19]. These include microbial metabolites such as 12,13-dihydroxyocatadecanoic acid (12,13-diHOME), short chain fatty acids (SCFAs), and trimethylamine (TMA), as well as microbial-host co-metabolites, such as taurine, polyamines, and hippuric acid. Many of these metabolites have only recently been identified in human milk, and their relationship to infant health is unknown. While there is an assumption that these metabolites derive from the maternal circulation (potentially originating from the maternal gut microbiome or maternal metabolism), local production of metabolites by the mammary cells and the milk microbiota is also likely [20,21]. The potential of some of these small metabolites to modulate infant health is already apparent. For example, human milk 12,13-diHOME has been associated with both lowered subcutaneous fat and slower body mass index (BMI) increase in early life, linking milk 12,13-diHOME to obesity [20]. Additionally, there is a correlation between infant fecal 12,13-diHOME and eczema and allergy, demonstrating the immune programming potential for this human milk metabolite [22]. Similarly, human milk SCFAs have also been linked to infant weight gain [23] and immunity [21]. Due to the causal role of the microbiome in many adult diseases, microbes and their small metabolite products are of high interest in early life, with human milk potential serving as a constant source of these important products [12].

**Figure 1:**
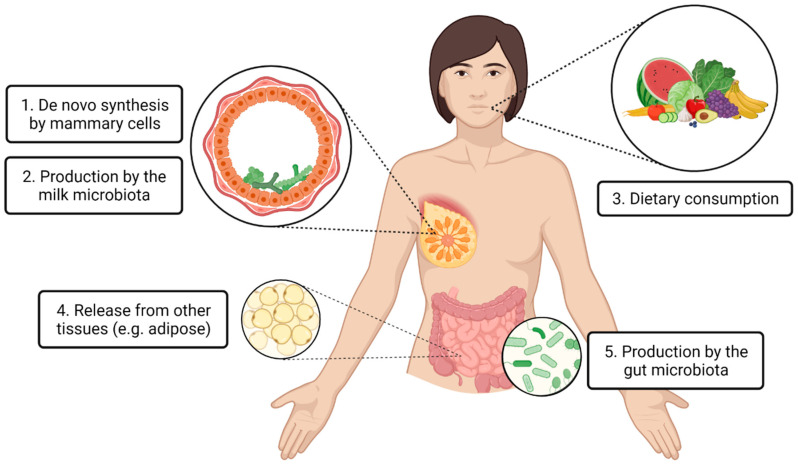
Proposed origins of lipids and small metabolites found in human milk. Local production within the mammary gland (**1**,**2**) and transport from distal body locations via the circulatory system (**3**,**4**,**5**) are hypothesized.

To the best of our knowledge, the latest article reviewing similar content was published in 2013, focusing on the impact of maternal nutrition and genetics on human milk fatty acids [24]. Since then, many other lipids and small metabolites have been identified in human milk, and vast advancements have been made in human milk research techniques, including sample collection and storage [25], DNA and lipid extraction [26,27,28], mass spectrometry and nuclear magnetic resonance [29,30], DNA sequencing [31], and cell sequencing [32]. Thus, there is a need currently to review recent evidence regarding the origins of human milk lipids and small metabolites. With the uprise of omics research in the human milk field, it is critical that we consider the origins of milk lipids and small metabolites, as well as other components of milk. Importantly, such an understanding feeds into the framework of human milk as a complex biological system, recognizing its relationship with other maternal body sites and systems [33]. This will allow better interpretation of findings, and better application of research to translation. The purpose of this review is to present the existing evidence on the origins of human milk lipids and small metabolites to inform future research directions and application. A high degree of variability in lipid and small metabolite concentrations has been identified across milk samples from different individuals around the globe [14,34], suggesting that determination of milk composition is complex and dynamic. Human milk lipids and small metabolites may be synthesized locally within the mammary gland (by the mammary cells or the milk microbiota) or they may be incorporated from maternal circulation (originating from the maternal diet, maternal gut microbiota, or other non-mammary tissue) (Figure 1). The evidence for each of these pathways will be discussed.

## 2. Local Production within the Mammary Gland

### 2.1. De Novo Synthesis by Mammary Cells

During lactation, the human mammary gland is one of the most metabolically active organs, with many synthetic processes unique to mammary cells, or upregulated to a degree much higher than other lipogenic organs (such as the liver). Of particular interest, the capacity to esterify fatty acids to glycerol, forming triacylglycerols, and to position over 70% of the C16:0 in the sn−2 position, are quite unique to the mammary gland [35,36]. High gene expression has been measured from cells in human milk during lactation, including genes for lipolysis, fatty acid uptake, de novo fatty acid synthesis, elongation and saturation of fatty acids, and triacylglycerol and cholesterol synthesis [35].

De novo fatty acid synthesis occurs in the mammary cell cytosol, utilizing glucose and NADPH substrates, and fatty acid synthase. Thioesterase II is highly expressed only in mammary cells, and specifically controls the chain length of these fatty acids, uniquely favoring medium chain C14:0 [37]. Further to this, de novo mammary synthesis of C16:0 to C18:1, and its control, remain unclear. Much of the understanding of de novo synthesis is based on animal models, and mouse studies have indicated that between 15 and 40% of milk fatty acids are a result of de novo synthesis [38]. Evidence from fatty acid synthase knock-out mice demonstrates the importance of de novo mammary synthesis, with knock-out dams producing milk low in fatty acids and experiencing early involution, resulting in pups who cannot thrive [36]. Human evidence on de novo mammary lipid synthesis is scarcer due to methodological difficulties. In place of gene knock-out experiments, human studies rely on the estimation of fatty acid synthesis based on measurements of dietary intake and stored fatty acids. Regardless, a small number of human studies have suggested approximately similar portions in mice and humans. Data from one human tracer study, with only three participants, indicate that de novo fatty acid synthesis provides 10–12% of milk fatty acids [39].

Following de novo synthesis of fatty acids, they are esterified to produce triacylglycerols, phospholipids, and other lipids, through glycerol−3-phosphate and monoglyceride pathways [40]. Transcriptomics on mammary cells isolated from human milk has shown expression of many lipid metabolism genes, including desaturases (FADS 1–6) and elongases (ELOVL 1–6) suggesting that the mammary gland has the capacity to synthesize all types of fatty acids, including long chain polyunsaturated fatty acids, which have commonly been thought to originate from diet. Other lipid types may also be synthesized by mammary cells, including gangliosides and phosphatidylcholine, based on expression of ST3GAL5, which encodes GM3 synthase, and CHKA and CHKB, which encode choline kinase alpha and beta [32]. Increasing accessibility to rapidly advancing techniques in single cell omics, allowing identification of novel gene expression and synthesis pathways, will facilitate further understanding on this front; however, results from milk-derived cells should be interpreted with some caution, as they may not be entirely representative of in vivo mammary cells [32,35,41].

### 2.2. Production by the Milk Microbiota

Human milk harbors a low biomass, low diversity, viable bacterial microbiome, predominantly comprising Staphylococcus and Streptococcus species, along with lower abundances of typical oral, skin, and gut taxa, such as *Cutibacerium*, *Gemella*, and *Bifidobacteria* [42]. The milk microbiome varies both between individuals (in relation to factors such as delivery mode, parity, and maternal BMI [43]), and within individuals over time [44,45]. This community may actively produce lipids and metabolites within the mammary gland; however, to date, limited evidence supports this notion. Whether the bacteria detected in human milk represent a permanent mammary gland microbiome, or whether they are transiently present is still unconfirmed [42]. This is worth noting, as the nature of the milk microbiome has implications for our understanding of the metabolic activity of the milk microbiota within the lactating mammary gland. Bacterial metabolites are present in human milk [12,21,34,46,47], although it is currently unclear whether they are produced locally in the mammary gland by bacterial metabolism, or whether they are derived from the maternal gut microbiota (see Section 3.3, Production by gut microbiota). For instance, short chain fatty acids (SCFAs) are present in human milk [21,46] at concentrations and ratios that differ vastly from those seen in serum (Table 1) [48,49], potentially suggesting local production or degradation within the mammary gland. Indeed, propionate, which is present in serum at concentrations similar to butyrate [49] and has been detected in the blood of pregnant individuals [50], has only recently been detected in human milk and appears to be present in negligible concentrations compared to butyrate [51]. Bacteria that possess the capacity to produce SCFAs from metabolism of human milk oligosaccharides (HMOs), such as Bifidobacterium, are present in human milk [42]. However, SCFAs are also present in maternal serum [48], suggesting that gut-derived SCFAs may be distributed into milk via the circulation. To date, no direct evidence has demonstrated that HMOs are metabolized in situ in the lactating breast. The origins of human milk SCFAs therefore remain unknown.

Data on the metatranscriptome of human milk suggest that the bacteria within the mammary gland actively transcribe genes for biosynthesis of various compounds, including secondary metabolites, such as amines and chorismic acid, and aromatic compounds, such as phenols and indoles [59]. Given that human milk bacteria are transcriptionally active, and that substrates for bacterial metabolism (such as HMOs and glucose) are present in human milk, it is plausible that local production of bacterial metabolites occurs in the lactating mammary gland. While bacterial lipid synthesis pathways are well-described [60,61], uniquely bacterial-derived lipids are yet to be reported within human milk. Evidence from transcriptomic work has not yet described active transcription of microbial lipid synthesis genes in human milk. However, this is likely due to the limited number and nature of such studies rather than lack of such synthesis. Targeted polymerase chain reaction (PCR) for bacterial lipid synthesis genes may overcome this gap in the literature.

## 3. Incorporation from Maternal Circulation

Many components reach the mammary gland and are incorporated into human milk from the maternal circulation. During the colostrum phase, lipids and small metabolites from circulation may cross into milk via the paracellular pathway (free transport via openings between epithelial cells); however, following closure of the tight junctions between lactocytes and transition to mature milk, transport must occur via transcellular pathways (through epithelial cells) [62]. From the bloodstream, lipids and small metabolites, including approximately 60% of the utilized fatty acids [39], are either packaged or further metabolized in the mammary gland, ending up in the milk fat globule for secretion [62]. Metabolite transport mechanisms across the mammary epithelium likely depend on the size and properties of the metabolite, as well as other conditions, and may include pinocytosis-exocytosis (which has been demonstrated for molecules up to ~320 kDa) or receptor-mediated transport [62,63]. The uptake of many lipids from circulation by mammary cells has been demonstrated in labeled animal studies, where lipoprotein lipase presence allows uptake of fatty acids, cholesterol, and phosphatidylcholine in rats [64]. While this has not been demonstrated in humans, lipase increases dramatically at lactation initiation, with increasing milk production [65]. Further, many fatty acid transporter genes are expressed in mammary epithelial cells, allowing fatty acid uptake via transmembrane proteins such as FAT and FATP, although this remains an understudied process [35]. Understanding of transport of lipids from the circulation to the lactating mammary gland is also important, given evidence that these mechanisms may be disrupted in cases of obesity and low milk supply [66].

### 3.1. Dietary Consumption

Maternal diet has been most strongly linked to milk fatty acid composition, with the assumption that dietary intake reaches circulation and is transported to the mammary gland. It is estimated that 29% of fatty acids originate from the diet and thought that the high degree of variability in milk fatty acid composition between women is a reflection of dietary variation [39,67]. This is of particular interest as it implies that dietary modulation may affect human milk lipid composition, with potential effects on the health of breastfed infants. For instance, maternal consumption of linoleic acid leads to a spike in linoleic acid concentration in maternal milk 8–24 h later [68]. Similarly, maternal intake of docosahexaenoic acid (DHA) is strongly correlated to milk DHA [69,70], and many milk fatty acids have been shown to be positively correlated with maternal circulating lipids, including arachidonic, oleic, and linoleic acids [71]. However, studies reveal that this is not entirely clear; it appears that both habitual diet and acute diet can impact human milk fatty acid composition to varying degrees [67,72,73]. Tracer studies are used to investigate the incorporation of dietary fatty acids into human milk, which involve consumption of labeled fatty acids and subsequent measurement of their presence in human milk and blood over many hours following consumption. These studies are often limited by small sample sizes, population spread, and restrictive maternal diets; however, the proportion of fatty acid incorporation from diet in humans seems to match that of other animals relatively closely; therefore, the mechanisms involved in this process may be comparable across mammalian species [39].

Small intestine absorption of metabolites consumed in the diet varies depending on the nature of the metabolite. Typically, water-soluble species (such as carbohydrates and proteins) are able to enter the blood stream, while fat soluble species (such as lipids and some small metabolites) predominantly enter the lymphatic system through lacteals and are able to then enter the bloodstream via the thoracic vessel to the heart [74]. This means that, when ingested, the most water-soluble species (for example, SCFAs) may enter the bloodstream directly from the small intestine and reach the mammary gland via the circulation, whereas uptake of lipid and lipid-soluble species may occur via lymphatic absorption from the gut. Notably, very little research has characterized metabolite transport from the gut to the lactating mammary gland, with the entero-mammary route remaining relatively speculative [75].

### 3.2. Release from Other Tissues

Non-mammary tissues (such as liver and adipose tissue) also synthesize, store, and release lipids and small metabolites into circulation. Study of maternal intake of labeled fatty acids has indicated that body stores are the major source of many fatty acids in human milk [72,73]. In one such study, while approximately 88% of arachidonic acid (AA) did not originate from the diet, only a very small portion (~2.2% of the total milk AA) was synthesized by non-mammary tissue linoleic acid conversion, confirming that the remainder originated from non-mammary tissue stores [72]. An estimated 59% of milk fatty acids originate from non-mammary adipose tissue [39]. Another example is 12,13-diHOME, a linoleic acid metabolite that is released from brown adipose tissue following cold exposure or exercise [55,76]. Following moderate maternal exercise, 12,13-diHOME concentrations in human milk increase 1.4-fold in 90-min [20]. Incidentally, 12,13-diHOME is also of interest as a bacterial metabolite [12] and has been linked to infant adiposity and immune programming outcomes [20,77].

### 3.3. Production by Gut Microbiota

The maternal gut microbiota produces a slew of lipids and small metabolites, including SCFAs via fiber metabolism [60,61,78], which elicit both local and systemic effects [78,79,80]. One example of a gut microbiome metabolite that has recently garnered interest in the human milk field is 12,13-diHOME. This metabolite is an end product of linoleic acid metabolism. 12,13-diHOME production is encoded by epoxide hydrolase genes, which are present in human, bacterial, and fungal genomes [22,81,82,83]. While it is unclear the extent to which milk 12,13-diHOME is produced by human or microbial genes, bacterial epoxide hydrolase genes have been reported to be present in the infant gut microbiome [22]. Human milk and infant gut 12,13-diHOME have recently been linked to infant body composition [20] as well as infant allergy and asthma development [22,84]. Thus, bacterial metabolites in human milk are currently of great interest for infant health.

Gut microbial metabolites can be detected in maternal blood [48] and may therefore be transported to the lactating mammary gland (Table 1). For instance, SCFAs produced by gut bacteria are first absorbed into colonocytes, where they are used as a local energy source (primarily butyrate). Unused SCFAs then enter the liver via the portal vein where they are further utilized. Thus, only a small portion of overall SCFAs reach the maternal circulation and have the opportunity to be incorporated into the mammary gland [85,86]. However, transport of gut bacterial metabolites from the maternal circulation to milk is understudied, with little evidence regarding the extent to which they are able to cross the mammary epithelium. This is a critical gap in the understanding of the milk metabolome. Given the well-documented health-modulating effects of microbial metabolites [87,88,89,90], it is important to understand the extent to which maternal gut metabolites may integrate into milk and be ingested by the infant. Importantly, this may open opportunities to modulate the maternal gut microbiome to improve infant health. To better understand transport of gut microbial metabolites to human milk via the circulation, paired blood and milk samples should be analyzed to identify correlated metabolites. This work has been performed in dairy cows, finding that milk is a distinct metabolic compartment with a composition largely independent from that of plasma, with the exception of a small number of key metabolites that were found to be tightly correlated between plasma and milk. These correlations include the bacterial metabolite trimethylamine (TMA) and the host-bacterial co-metabolite dimethyl sulfone (DMSO_2_) [91] and suggests selective transport of certain metabolites from the maternal circulation into milk. As similar studies are lacking in humans, we have synthesized data from a variety of human studies to assess levels of key gut bacterial metabolites in the circulation compared to milk (Table 1). These data highlight that the human milk metabolome is distinct from that of blood, suggesting selective transport of bacterial metabolites across lactocytes. However, it should be noted that data on blood metabolite levels are sourced from the general adult population. Analysis of paired blood and milk samples from lactating mothers is necessary to verify these relationships.

## 4. Summary and Future Research Directions

Given the myriad of health effects of lipids and small metabolites for the breastfed infant [11,12], they are promising therapeutic targets to improve infant health. However, without an understanding of the origins of milk metabolites and lipids, we are limited in our ability to modulate these components. For instance, an understanding of the degree to which various maternal dietary lipids are able to reach the mammary gland may underpin future dietary recommendations for breastfeeding women. Here we have highlighted five sources for milk lipids and metabolites: de novo synthesis from mammary cells, production by the milk microbiota, dietary consumption, release from non-mammary tissue, and production by the gut microbiota. In order to further elucidate the origins of lipids and metabolites in human milk, we propose the following research strategies.

### 4.1. Transcriptome Analysis of Human Milk Cells

There is limited transcriptional data on human milk cells, due to the challenge involved in acquiring appropriate samples and the cost associated with such analyses. While uptake of such work is increasing in the field [32,35,92,93], further work is needed to define synthesis pathways for milk lipids and metabolites. Further analysis of transporter gene expression in mammary epithelial cells will also aid in defining transcellular transport mechanisms for circulating lipids and metabolites. It is important, however, to identify how closely cells isolated from milk represent those that produce milk in vivo, as prior data suggest important differences. Thus, other techniques are likely required, such as organoid modeling.

### 4.2. Paired Sample Analyses

It is necessary to analyze paired blood and milk samples from lactating individuals in order to better understand the degree to which circulating lipids and small metabolites are transported into the lactating mammary gland. While comparison of milk metabolite concentrations to circulating metabolite concentrations in non-lactating individuals suggests that the mammary gland is a distinct metabolic compartment, further paired analyses are needed to verify this finding. A recent example of a comprehensive study attempting to elucidate human milk component origins utilized milk, blood, and adipose tissue, and further solidified the evidence of milk fatty acids originating from adipose tissue [65]. Future studies could include other matched samples such as fecal samples, advanced dietary data collection and analysis, and labeling technologies, to add insight into component origin and final destination.

### 4.3. Dietary Studies

Many of the metabolites discussed in this review are derived from bacterial metabolism of host dietary components, highlighting the need to couple metabolomic and microbiome analyses with dietary intake data. Advanced methods for dietary data analysis continue to emerge and will benefit this work greatly [94], particularly if labeled studies are incorporated [95], to improve our understanding of the impact of maternal diet on the breastfed infant.

### 4.4. Bacterial Gene Analysis

A current gap in the literature is the inability to identify whether microbial metabolites or lipids in human milk are produced by the gut or milk microbiota. Targeted PCR for known bacterial metabolite/lipid synthesis genes would demonstrate the capacity of milk bacteria to produce these components. Whole genome analysis would achieve a similar outcome but is made difficult by the low biomass of the milk microbiome and the relatively high portion of host DNA [31,96]. However, transcriptional analysis is necessary to demonstrate gene activity (and not just gene presence). Similar to whole genome sequencing, metatranscriptome analysis of the human milk microbiome is limited by biomass issues [31,96]. Targeted RNA-sequencing of genes of interest or use of reverse transcription PCR (RT-PCR) may therefore be necessary; however, such techniques require prior understanding of genes involved [97,98].

## 5. Conclusions

Human milk lipids and small metabolites are a critical component of human milk, with roles in infant growth, immunity, and development. Despite this, many gaps remain as to the origins of these, and thus translation of compositional research to improve infant health is hampered. Continued research into the maternal and microbial origins of human milk lipids and small metabolites is critical, and all compositional research should be conducted with the potential origins of components in mind.

## Figures and Tables

**Table 1:** Key health-modulating gut bacterial metabolites that have been identified in human milk. Previously reported levels from blood and milk samples are provided. Values are mean ± SD, or mean (range), depending on the reporting style of the study. Concentrations are µM unless stated otherwise.

Metabolite	Origin	- Host Health Effects	Mean Level in Circulation	Mean Level in Milk	References
Short chain fatty acids (acetate, butyrate, formate)	Bacterial fermentation of polysaccharides	- Inhibition of inflammatory cytokines - Promotion of differentiation and expansion of regulatory T cells - Promotion of gut barrier integrity - Appetite regulation - Lipid metabolism - Glucose homeostasis - Epigenetic modifications	Acetate: 41.9 ± 15.1 Butyrate: 1.0 (0.3–1.5) Formate: 32.8 ± 13.3	Acetate: 27.5 ± 28.2 Butyrate: 192 ± 149 Formate: 12.2 ± 14.8	Milk: [46] Blood: [48,49]
Polyamines (putrescine, spermidine, spermine)	Arginine derivative produced by host and bacterial cells	- Inhibition of inflammatory cytokines - Maintenance of intestinal mucosa and resident immune cells - Regulation of transcription, translation, and post-translation modification - Cell proliferation, differentiation, and apoptosis - Regulation of ion channel function	Putrescine: 0.05 ± 0.03 Spermidine: 0.07 ± 0.04 Spermine: 0.03 ± 0.04	Putrescine: 0.21 (0–1.14) Spermidine: 3.53 (1.43–6.47) Spermine: 5.08 (0.76–5.94)	Milk: [52] Blood: [53]
12,13-DiHOME	End metabolic product of linoleic acid produced by host, fungal, and bacterial cells	- Regulation of brown adipose tissue fuel uptake and thermogenesis - Alteration of peroxisome proliferator-activated receptor γ regulated gene expression in dendritic cells - Reduction in regulatory T cells - Suppression of IL−10 secretion in dendritic cells	0.0012 ± 0.00018	0.0056 ± 0.0004	Milk: [54] Blood: [55]
Trimethylamine	Bacterial metabolism of choline	- Precursor to Trimethylamine N-Oxide (TMAO), which has been linked to insulin resistance, fatty liver disease, and atherosclerosis	0.418 ± 0.124	<5 ppm	Milk: [56] Blood: [57]
Hippurate	Bacterial metabolism of polyphenols and host conjugation from glycine and microbial benzoate	- Negatively associated with numerous metabolic conditions, including blood pressure, non-alcoholic fatty liver disease, visceral fat mass and Crohn’s disease	16.74 ± 11.16	7.8 ± 4.2	Milk: [46] Blood: [53]
Taurine	Bacterial deconjugation of taurine from host taurine-bound bile acids	- Antioxidant action - Anti-inflammatory action - Restoration of fatty acid oxidation - Conjugation of bile acids to facilitate lipid absorption - Modulation of gene expression - Attenuation of endoplasmic reticulum stress - Osmoregulation - Promotion of Ca^2+^ homeostasis	93.0 ± 35.7	190 ± 66.6	Milk: [46] Blood: [58]
